# Aerolysin Nanopore Structures Revealed at High Resolution in a Lipid Environment

**DOI:** 10.1021/jacs.4c14288

**Published:** 2025-02-03

**Authors:** Jana S. Anton, Ioan Iacovache, Juan F. Bada Juarez, Luciano A. Abriata, Louis W. Perrin, Chan Cao, Maria J. Marcaida, Benoît Zuber, Matteo Dal Peraro

**Affiliations:** † Institute of Bioengineering, School of Life Sciences, École Polytechnique Fédérale de Lausanne, 1015 Lausanne, Switzerland; ‡ Institute of Anatomy, University of Bern, Baltzerstrasse 2, 3012 Bern, Switzerland; § Department of Inorganic and Analytical Chemistry, Chemistry and Biochemistry, University of Geneva, 1211 Geneva, Switzerland

## Abstract

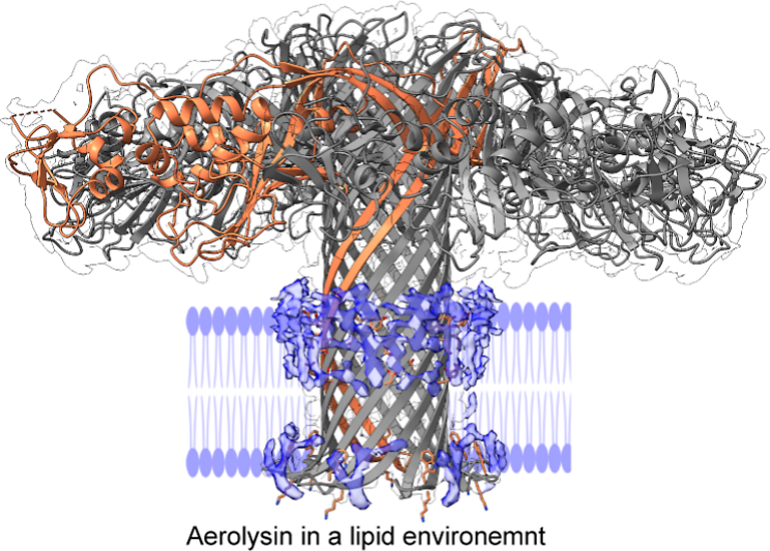

Aerolysin is a β-pore-forming toxin produced by most Aeromonas bacteria, which has attracted large attention in the field of nanopore sensing due to its narrow and charged pore lumen. Structurally similar proteins, belonging to the aerolysin-like family, are present throughout all kingdoms of life, but very few of them have been structurally characterized in a lipid environment. Here, we present the first high-resolution atomic cryo-EM structures of aerolysin prepore and pore in a membrane-like environment. These structures allow the identification of key interactions, which are relevant for understanding the pore formation mechanism and for correctly positioning the pore β-barrel and its anchoring β-turn motif in the membrane. Moreover, we elucidate at high resolution the architecture of key pore mutations and precisely identify four constriction rings in the pore lumen that are highly relevant for nanopore sensing experiments.

## Introduction

**Figure 1:**
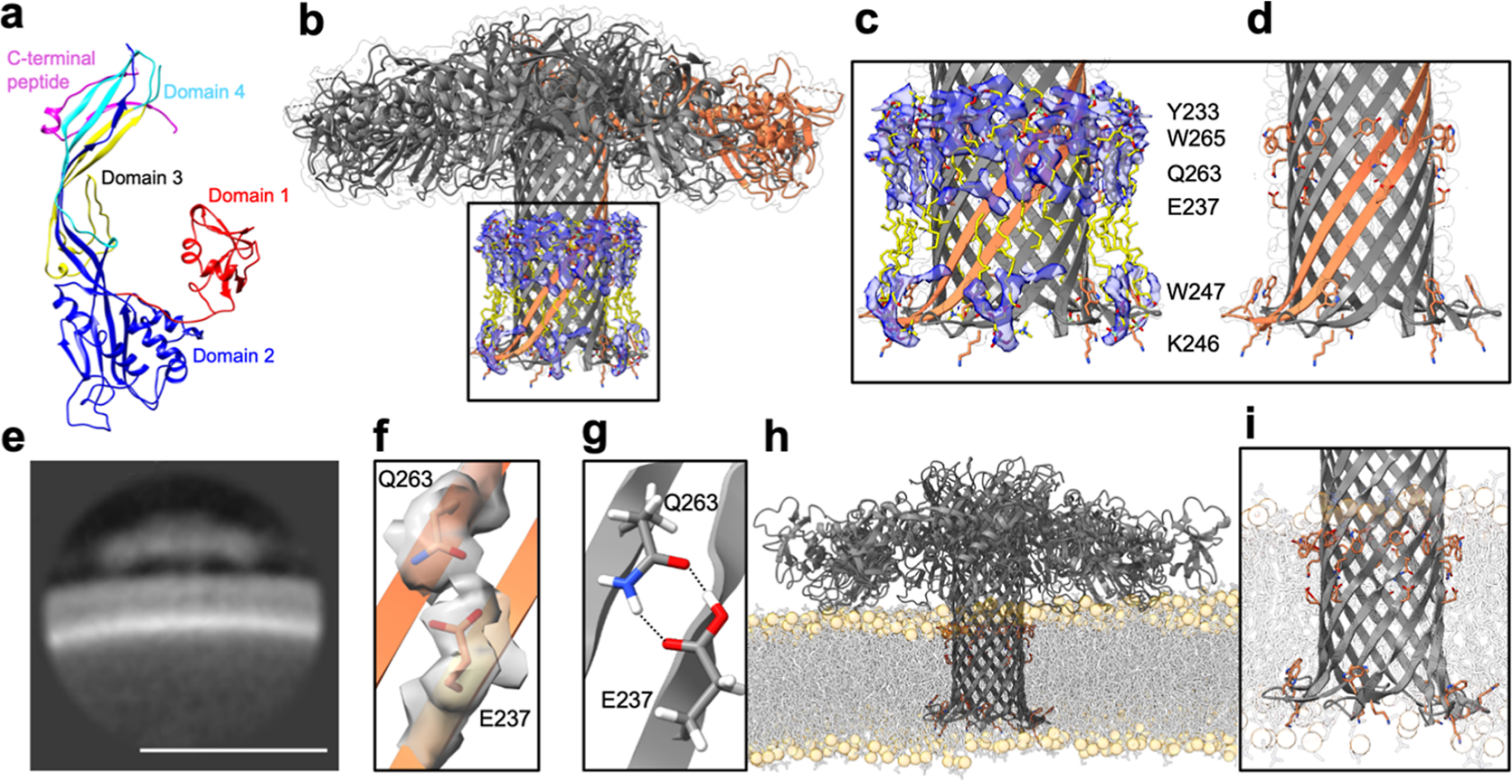
| High-resolution structures of wild-type (WT) aerolysin in styrene maleic-acid lipid particles (SMALPs). (a) Structure of proaerolysin (PDB ID: 1PRE). Domain 1 is colored in red, domain 2 is colored in dark blue, domains 3 and 4 are colored in turquoise for the part that forms the concentric β-barrel and in yellow for the part that forms the stem β-barrel. The C-terminal peptide is colored in pink. (b) Structure and density map of aerolysin in SMALPs (all other structures are shown in Supporting Information Figures S1–S7). Extra density is highlighted in blue. One protomer and membrane lining residues are highlighted in orange. Lipids are modeled into the extra density and shown in yellow. (c,d) Zoom into the transmembrane part of the aerolysin barrel with and without extra densities observed in the SMALP data set. Extra density is shown in blue and the modeled lipids in yellow. Membrane lining residues are highlighted in orange. (e) Example of a 2D class of aerolysin in DOPC/DOPE (2:1) liposomes. The bar represents 150 Å. (f) Interaction between Q263 and E237 in the cryo-EM structure shown with density map. (g) Interaction of Q263 and E237 in the MD simulation. (h) Snapshot from atomistic MD simulation of the aerolysin pore equilibrated in a DOPC/DOPE (2:1) bilayer mimicking the nanodisc conditions. The phosphates of the lipids are highlighted as spheres. Membrane lining residues are highlighted in orange. (i) Zoom into the transmembrane barrel of aerolysin in the MD simulation.

Pore-forming toxins (PFTs) are important virulence factors of many pathogenic bacteria, which are secreted in a soluble form and upon host membrane binding, they oligomerize into transmembrane pores undergoing a complex structural reorganization.^1^ Aerolysin, a major virulence factor of *Aeromonas hydrophila*, is one of the best characterized β-PFTs.^1−3^ The structure of the soluble form of aerolysin was solved 30 years ago by X-ray crystallography.^4^ The 52 kDa monomer of aerolysin is composed of four domains with different roles in its mode of action (Figure 1a): the first two N-terminal domains (domains 1 and 2) are required for receptor binding; the domain 3 forms most of the transmembrane pore; and the domain 4, which is the most C-terminal domain, functions as a chaperone and mediates pore formation.^5−7^ While the structures of the intermediate oligomers have been solved by cryo-EM at medium resolution (3.5–4.5 Å), the structure of the mature aerolysin pore has been so far elusive.^8^ Only a crude model of the pore in detergent micelles has been obtained from a low-resolution map (∼8 Å),^8^ revealing a concentric double β-barrel structure, which is a novel and unique folding motif among known PFTs.

Since then, aerolysin has gained particular interest in the field of nanopore sensing due to the unique structural and electrostatic features of its narrow inner pore.^9,10^ In nanopore sensing, the specific nature of an analyte is determined by analyzing its current signature and residence time as it translocates through a pore.^11^ The long, narrow, and charged lumen of aerolysin is characterized by two distinct constrictions narrower than one nanometer. It has enabled the detection and analysis of diverse biomolecules and the discrimination of single amino acids, nucleotides, and glycans.^12−16^ The introduction of mutations in aerolysin has been shown to increase its sensitivity. For instance, the K238A mutation leads to a large increase in the selectivity and sensitivity of the pore presumably by modifying the size of its constrictions.^13^ Also other mutants, such as K238A-K244A, R220A, and T240R, have shown improved sensing capabilities by modifying one of the two constriction sites of the pore.^16,17^ However, a high-resolution structure of aerolysin is currently missing, hindering a more precise understanding of its biological function and sensing capabilities.

Here, we present the first high-resolution cryo-EM maps and atomic models of WT aerolysin in a membrane-like environment and its Y221G prepore-like mutant. In addition, we also solve some key mutants that are regularly used in nanopore experiments, namely, K238A and K238A/K244A (Figures 1 and S1–S7 Supporting Information). These high-resolution structures allow the identification of important interactions required for pore formation and reveal four constriction rings in the pore lumen.

## Results and Discussion

### CryoEM Structure of Wild-Type Aerolysin in Multiple Membrane Mimics

The type of membrane mimetic used to solubilize membrane proteins can affect protein structure.^18,19^ To address this issue in the context of β-barrel proteins, we decided to compare the pores obtained in lauryl maltose neopentyl glycol-cholesteryl hemisuccinate (LMNG-CHS), amphipols,^20^ as well as SMALPs.^21^ We obtained near-atomic resolution (2.2 Å) cryo-EM maps of WT aerolysin solubilized in either amphipols or SMALP, as well as in LMNG-CHS (2.6 Å) (Figures 1b and S1 Supporting Information). Interestingly, we did not observe any major differences between the density maps and models derived from them (Supporting Information Figure S1 and Tables S1 and S2). This result may seem to contradict previous results that have shown that different solubilization methods impact membrane protein structures in different ways.^19^ However, β-PFTs are highly stable β-barrel structures, and all methods of sample preparation that we tested seem equally suited for their structural characterization, a property that can likely translate to all symmetric oligomeric β-barrel proteins. Additionally, a data set of aerolysin pores in liposomes was collected. The 2D classes derived from this data set confirm the stable positioning of aerolysin in the membrane and look very similar to 2D classes obtained from the SMALP, amphipol, and LMNG-CHS data sets (Figure 1e).

The overall Cα root-mean-square deviation (RMSD) between the structures in amphipol and SMALP is 0.82 Å for the overall structures and 0.74 Å for the extended β-barrels region (i.e., residues 215–285) (Supporting Information Tables S1 and S2). Differences between the previously proposed aerolysin pore model and the new high-resolution structures of aerolysin can be observed particularly in the orientation of side chains and loops. The overall RMSD between the new structures in nanodisc/amphipol and the previous model is ∼3 Å (∼2.5 Å RMSD considering only the extended β-barrel).

The aerolysin pore shows a mushroom-shaped structure with the cap lying parallel to the lipid bilayer, as previously reported.^8^ The density for the outer loops of the cap (residue 15–24), which are involved in receptor binding, are less resolved in the cryo-EM maps, and a 3D variability analysis on the cryoEM data indicates a larger flexibility at this region (Supporting Information Video S1).

Domains 3 and 4 form the 120 Å long 14-stranded β-barrel, with each protomer contributing two antiparallel β-strands (Figure 1b). The transmembrane pore segment has unclear hydropathy features, so its proper position in the membrane is not as obvious as that for other proteins. However, the new high-resolution structures in membrane mimics allow a better determination of the interactions of aerolysin with lipids as well as the precise positions of amino acid side chains. The cryoEM map in SMALPs shows an additional density, not observed with amphipol, which allows to partially model some of the lipids surrounding the β-barrel (Figure 1c,d). At the bottom, the β-barrel terminates in a partially hydrophobic β-turn motif (amino acids 246–251, Figure 1c,d), which is tilted outward and is embedded into the membrane. Together, the seven β-turns form a motif that locks the pore in the membrane, i.e., the rivet structure previously predicted based on site-directed mutagenesis,^6^ later modeled,^8^ and now finally resolved at high resolution. Interestingly, W247, located at the bottom of the β-barrel, points toward the hydrophobic core of the membrane, anchoring the pore in the membrane as previously proposed. The only amino acid located in the rivet which points out of the membrane toward the polar lipid head groups is K246, providing a further anchoring mechanism (Figure 1c,d).

Additional density is observed in the transmembrane part of the β-barrel in the SMALP data set which cannot be observed in the amphipol data set. This extra density allows one to partially fit some phospholipids and allows one to pinpoint the position of the aromatic belt formed by Y233 and W265 (YW belt, Figure 1c,d) with respect to the membrane. This belt, a motif commonly observed in β-barrel proteins,^22,23^ anchors and stabilizes the β-barrel in the lipid membrane. The orientation of aerolysin in the membrane could additionally be confirmed by cryogenic electron tomography (Supporting Information Figure S8, Video S2), where the distance of the cap region from the membrane and the orientation of the pore can clearly be observed. Interestingly, just below the YW belt, two polar residues, E237 on one β-strand and Q263 on the opposite strand, interact with each other and are fully embedded at the core of the membrane hydrophobic environment (Figure 1b–d). The high resolution of our structures allowed us to define a strong interaction of E237 with Q263 which is highlighted by the continuous density between the two residues (Figure 1f). Carefully looking at the fitted model, such conformation could only be consistent with the formation of a double H-bond produced by a protonated E237, which is consistent with the expected higher p*K*_a_ of glutamates within the hydrophobic environment of the membrane.^24^ Interestingly, if one looks at the conservation of the E237-Q263 pair in aerolysin-like proteins (ConSurf^25^ analyzed at 95–50% homology threshold), Q263 is almost fully conserved (98%), while E237 is conserved only at 50% but replaced in the rest of the cases by a glutamine, which has equivalent H-bond features of a protonated glutamate. All this evidence clearly points to a protonated state for E237.

To further characterize the insertion of aerolysin into the membrane, we used MD simulations mimicking the cryo-EM conditions. We constructed an extended bilayer with the same lipid composition used in SMALPs (i.e., 2:1 DOPC/DOPE ratio), where we inserted the aerolysin pore model. The data accumulated in >300 ns of atomistic MD simulations globally recapitulates the features observed in cryoEM. The YW belt remains fully embedded into the lipid bilayer, and the aerolysin extracellular domains remain similarly positioned as in the cryoEM structure (RMSD = 0.9 Å when considering only the barrel region and 4.8 Å when considering the entire structure, Figure 1h,i). In detail, we notice how the rivet motif gets fully embedded into the bilayer with W247 deeply buried and only K246 able to snorkel out to the solvent phase, thus exerting the previously proposed riveting function. This uncommon conformation of the aerolysin pore could be the reason for the rectified behavior observed in nanopore experiments:^12,13^ K246 in fact is partially screened by the membrane interactions creating thus a not optimal condition for ion capturing. During the MD simulation, the pore barrel remains mainly perpendicular to the membrane, with limited fluctuation around this average orientation. We notice that only MD simulations set with protonated E237 are consistent with the cryoEM data and can preserve the strong E237–Q263 coupling. All MD simulations missing this key protonation state lose the interaction and produce an uplifted pore conformation within the membrane that leaves the YW belt out of the lipid environment (Supporting Information Figure S9). This result indicates the importance of high-resolution cryoEM data to confidently determine subnanometer structural features and to accurately model the mechanics of the pore in molecular simulations. Similarly, high resolution was key to more confidently model the protonation states of other important histidine residues (e.g., H132 and H186, see the Methods section).

### Structural Insights for the Pore-Forming Mechanism

**Figure 2:**
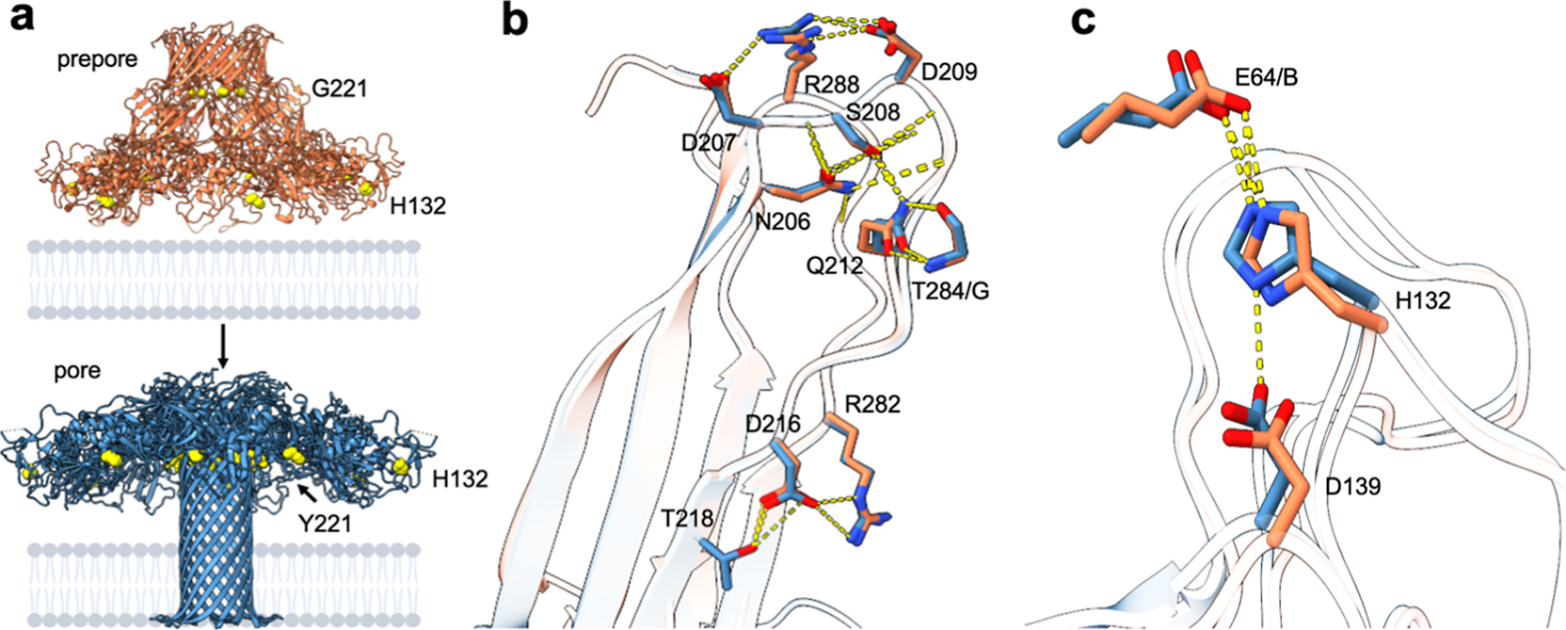
| Structural insights into the pore formation mechanism. (a) Overview of the structural changes from aerolysin prepore to pore. The residues H132, Y221, and G221 are highlighted as yellow spheres in the respective structures. (b) Overlay of aerolysin prepore and mature pore, zooming in on the concentric β-barrel fold where residues maintain polar interactions between side chains during the prepore to pore transition. Only protomer A is shown for both structures. Common bonds are formed between D207–R288, R288–D209, S208–Q212, and Q212–T284 of the neighboring protomer G and R282–D216. (c) Overlay of prepore and pore by the cap region highlighting the interaction of residues H132 with D139 and E64 of the neighboring protomer.

The extensive structural rearrangements that occur when aerolysin transitions from its prepore state (captured by the Y221G mutation^26^) to the mature pore have been previously described.^8,26,27^ This mechanistic model can be refined on the basis of our high-resolution cryo-EM structures as we also solved the structure of the prepore Y221G mutant at 1.9 Å resolution. The overall Cα RMSD with respect to the previous structure at 3.9 Å resolution (PDB ID: 5JZH) is 0.61 Å (Figures 2a, S7 and S10 Supporting Information). Upon prepore assembly, domains 1 and 2 of adjacent protomers interact through a series of salt bridges and H-bonds that greatly stabilize the cap region (Supporting Information Table S3). Furthermore, the formation of the concentric β-barrel fold contributes to the stabilization of the prepore through the addition of over 10 H-bonds per subunit. Interestingly, five H-bonds (Figure 2b) consolidate the lumen of the concentric β-barrel being present both in the prepore and pore configuration. The prepore stability is further enhanced by the hydrophobic residues at the interface of the two β-barrels.^8^ Upon membrane insertion, as previously described, the protein twists around two hinge regions flanking the third domain of the protein.^26^ Pore formation retains the tight network of bonds between protomers observed in the prepore, while introducing several additional interactions at the folded hinge regions (Supporting Information Table S3). Of note, the intersubunit interaction of H132 (which is predicted to be positively charged) and E64 confirms the previously identified critical role of H132 in oligomerization^28^ (Figure 2c).

### Geometry of the Pore Lumen and Implications for Molecular Sensing

**Figure 3:**
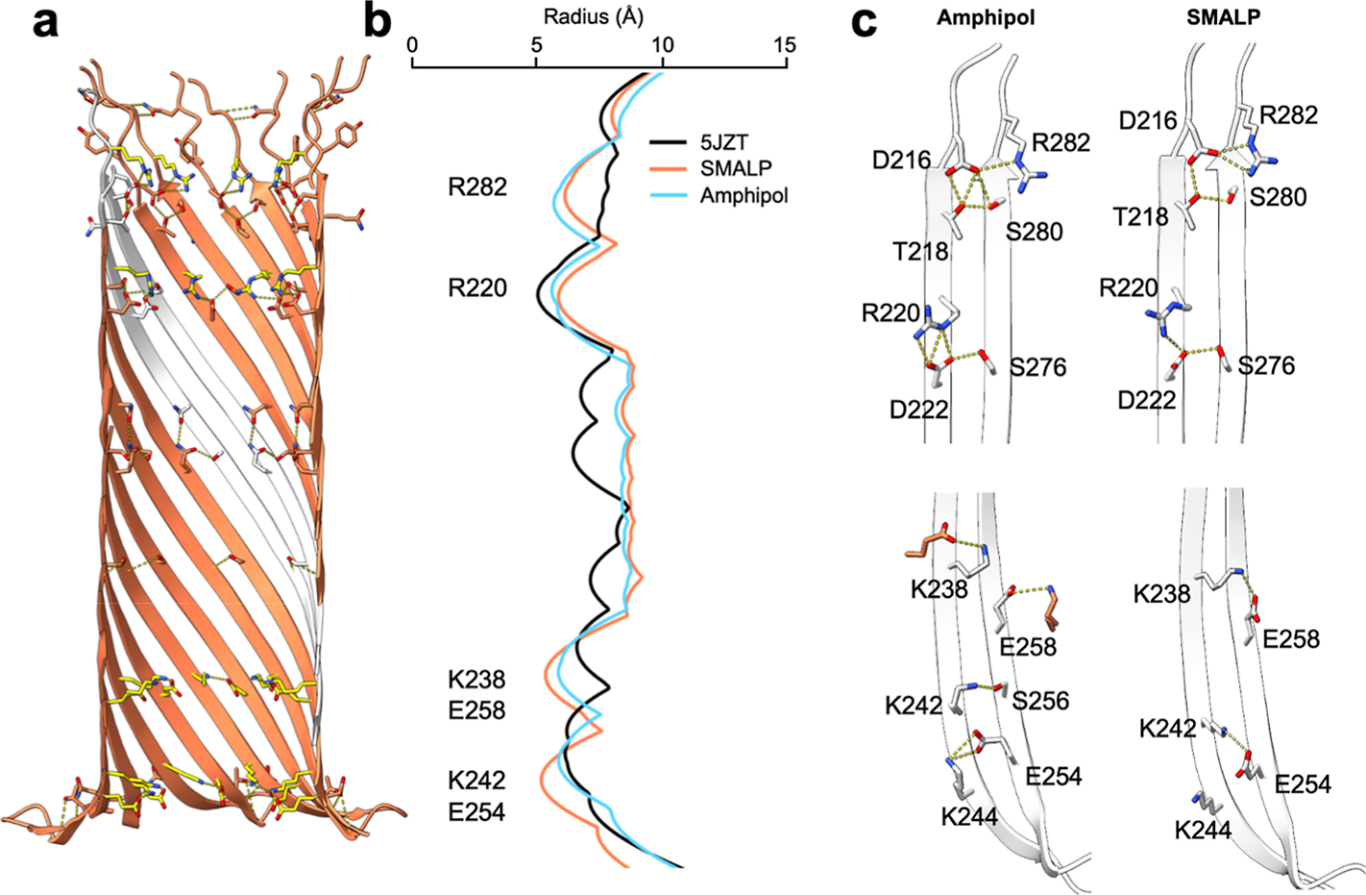
| Origin of aerolysin nanopore sensitivity. (a) Sideview of WT aerolysin lumen in SMALPs. Amino acids that form interactions on the side chain level in the pore lumen are shown as sticks. Amino acids causing constriction are highlighted in yellow. (b) Traces of the pore radii (Å) of the earlier low-resolution aerolysin structure (5JZT) (black), aerolysin in SMALP (orange), and aerolysin in amphipol (light blue). The radii traces were calculated with HOLE^29^ from the extracellular to the intracellular end of the pore lumen. (c) Comparison between the network of interactions found in the structures in amphipol and SMALP at the extracellular (top) and intracellular (bottom) ends of the pore.

The geometry of the pore lumen is of particular interest for nanopore sensing applications. Four distinct constriction rings, two at each end of the pore, were identified from the structures (Figure 3a,b) and confirmed by MD simulation (Supporting Information Figure S11). The two constrictions on the extracellular end of the β-barrel are caused by positively charged amino acids R282 and R220, respectively. R282 forms a salt-bridge with D216 on the same protomer which is involved in further interactions with T218 and S280 (Figure 3c). R220 forms polar interactions with D222, which in turn interacts with S276. At the cytoplasmic end of the transmembrane region, the other two constriction sites are defined by K238 and the K242/K244 pair. These lysines form a network of H-bonds and salt-bridges with E258, S256, and E254 on the antiparallel β-strand of the same or neighboring protomer.

Interestingly, this interaction network can differ between the WT pore in amphipol and in the nanodisc. In amphipol, K242 interacts with S256, while E254 interacts with K244; in the SMALP structure, K242 forms a salt-bridge with E254 (Figure 3c). These subtle differences in the interaction partners between the two structures suggest that the residues can switch between interaction partners on the same or neighboring protomer, thereby enhancing the stability of the barrel and establishing a robust geometry of the pore lumen. Overall, previously unseen interactions between side chain and backbone atoms can be observed in both prepore and pore conformations of aerolysin structures at high resolution.

The location of the constriction points is in agreement with the sensing spots previously proposed.^12,13^ The central region of the pore lumen exhibits a much wider cavity with a radius of ∼8–9 Å, while at the constriction sites, the size of the pore radius decreases to ∼5 Å. A comparison of the pore radius between the new structures and the previous aerolysin model revealed that only one out of the four constriction rings was clearly captured in the low-resolution model (Figure 3b). Similar observations were made when analyzing the water-accessible radius across the pore estimated during MD simulations (Supporting Information Figure S11). In particular, the role of R282 forming the outermost extracellular constriction ring is now more evident in the new structures while it was underestimated in the previous models (Figure 3b). This observation is of particular interest in the field of nanopore engineering where altering constriction size is thought to increase sensitivity.^13,16^

### CryoEM Structure of the K238A Aerolysin Pore Mutant

**Figure 4:**
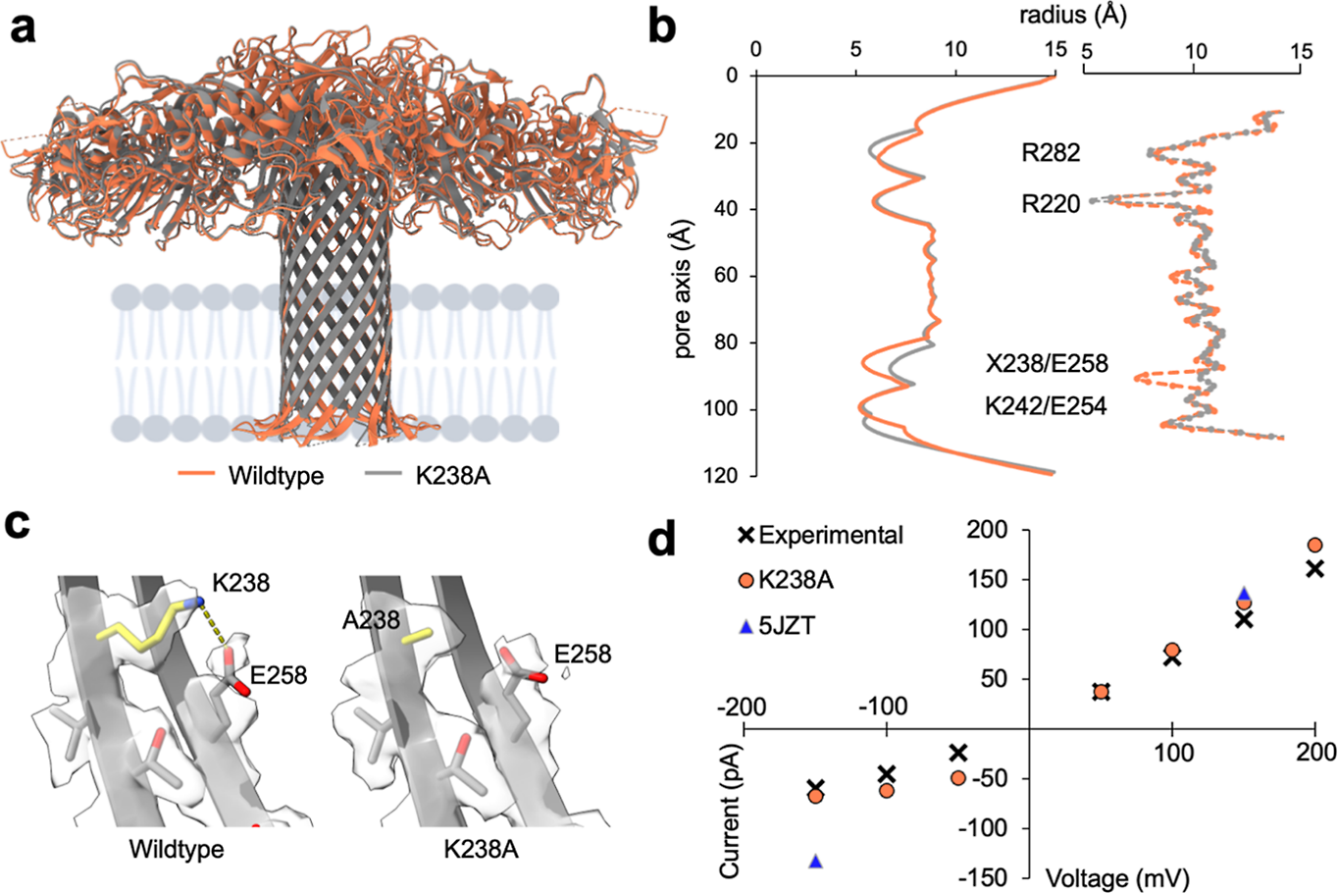
| Structure of the aerolysin K238A mutant used for nanopore sensing. (a) Overlay of WT aerolysin (orange) and aerolysin K238A (gray) in nanodiscs. (b) Comparison of the channel radii. On the left side, the radii is determined by HOLE.^29^ On the right side, the water accessibility radius across the pore is calculated from MD simulations. The amino acid in position 238 depicted by an X is a lysine in the WT and alanine in the K238A mutant. (c) Comparison of the interactions at the cytoplasmic end of the β-barrel in WT aerolysin (left) and the K238A mutant (right). The location of the mutation is highlighted in yellow. The density map is shown for residues 238–258. (d) Plot of the current at 1 M KCl as a function of voltage for the new K238A aerolysin structure (PDB ID: 9FNP) with a modified protonation state of E237, H132, H186 and H332 (see Methods for details), and previous K238A structure, which was obtained by mutating PDB ID: 5JZT using CHARMM-GUI. Experimental data at the same condition are shown as crosses,^12^ simulation results for the new K238A mutant are shown as orange circles and for the previous structure (PDB ID: 5JZT) as blue triangles.

We have recently demonstrated that the K238A mutation enhances the sensing capabilities and sensitivity due to a longer dwell time of the analyte in the pore.^13,30,31^ To unravel the structural basis for this improved performance, we have solved the structure of the K238A mutant in SMALP obtaining a density map at 2.2 Å. The structures of the WT and mutant in SMALP are highly similar, exhibiting an RMSD of 0.92 Å (Figure 4a) and comparable pore cavities shown both from the raw structure and in MD simulations (Figure 4b). Upon closer examination of the mutant structure, it becomes evident that the K238A mutation induces widening of the third constriction ring. This is attributed to the substitution of lysines with smaller alanine residue. In contrast to previous suggestions that the K238A mutation alters the analyte dwell time by decreasing the diameter of the constriction formed by R220,^13^ these structures reveal that all other constriction rings remain unaffected. Notably, for this mutant, we did observe a narrower water density from the MD simulation at R220, which is the same as previous MD results (Figure 4b). For analyzing the lowest constriction ring, we determined the structure of the K238A/K244A mutant in SMALP obtaining a resolution of 2.2 Å (Supporting Information Figure S12a). As expected, the two lysine to alanine mutations also increase the diameter of the lowest two constriction rings without affecting the upper constriction rings. Interestingly, the modification of only K244 leads to a decrease in the lowest constriction, indicating that both K242 and K244 are equally contributing (Supporting Information Figure S12). It has been previously reported that the change in sensitivity was not caused by the impact on the electrostatic potential by the mutation.^13^ The new structures indicate that the K238A mutation may influence the pore’s rigidity as it disrupts salt–bridge interactions between K238 and E258 within the barrel (Figure 4c).

In nanopore sensing experiments, the ionic current through the pore is used to determine the nature of the translocating analyte. A comparison of the ionic current response to different voltages revealed that the MD simulations in DPhPC bilayers based on the new K238A aerolysin structure solved in SMALPs yielded results that very closely aligned with the experimental data and are better than previous estimations, especially when negative voltage is applied (Figure 4d).^12,13^ We are confident, thus, that these new high-resolution structures will provide robust models for running future MD simulations supporting nanopore experiments.

## Conclusions

In conclusion, we report high-resolution structures of WT aerolysin along with its K238A and K238A-K244A mutants in SMALPs, amphipols, and LMNG. These findings enable us to refine the mechanistic understanding of aerolysin oligomerization and pore formation. We demonstrate that the structural integrity of a β-barrel transmembrane pore is retained across different membrane mimetics, and we reveal for the first time the position of the β-barrel with respect to the lipid environment. Moreover, our results explain the stability of the aerolysin pore and provide an atomistic view of the interactions conferring the exceptional resilience of the aerolysin pore to denaturation by heat or chaotropic agents.^32^ Of particular interest are the four constriction rings clearly identified in the pore lumen. The accurate structural characterization of these rings and the charge distribution within the pore lumen are of vital importance for the targeted engineering of aerolysin pores for tailored nanopore sensing applications. Therefore, our high-resolution structural data hold significant value for the future advancement in the fields of nanopore sensing and sequencing.^30,31,33,34^

## Methods

### Aerolysin Expression and Purification

WT and mutant aerolysins with a C-terminal hexahistidine tag were expressed using a pET22b vector in BL21 DE3 pLys *E. coli* cells. Cells were grown to an optical density of 0.6 to 0.7 in Luria–Bertani media shaking at 37 °C. Protein expression was induced by adding 1 mM isopropyl β-d-1-thiogalactopyranoside and subsequent growth at 20 °C overnight. Collected cell pellets were resuspended in lysis buffer (20 mM Sodium phosphate pH 7.4, 500 mM NaCl) mixed with complete Protease Inhibitor Cocktail (Roche) and subsequently lysed by sonication. After addition of 4 μL of TurboNuclease, the suspension was centrifuged at 20,000*g* for 30 min at 4 °C. The supernatant was applied to a HisTrap HP column (GE Healthcare) previously equilibrated with lysis buffer. Protein was eluted with a gradient over 30 column volumes of elution buffer (20 mM Sodium phosphate pH 7.4, 500 mM NaCl, 500 mM Imidazole). Fractions containing aerolysin were buffer-exchanged into aerolysin buffer (20 mM Tris, pH 8, 150 mM NaCl) using a HiPrep Desalting column (GE Healthcare). Aerolysin wt in SMALP was buffer exchanged into 500 mM NaCl, 20 mM Tris, pH 8. Purified protein was frozen in liquid nitrogen and stored at −20 °C until further usage.

### Liposome Preparation

For the liposome preparation DOPC and DOPE dissolved in chloroform were mixed in a mol ratio of 2:1 and dried down under a stream of nitrogen. Remaining solvent was removed by incubating the sample in a SpeedVac for 1 h at room temperature. Lipids were resuspended aerolysin buffer (150 mM NaCl, 20 mM Tris, pH 8) to a final concentration of 10 mg/mL. After at least five freeze and thawed cycle of the lipid solution, liposomes were formed by extrusion through a 100 nm filter using the mini-extruder from Avanti.

### Preparation of Aerolysin in SMALP

For the preparation of aerolysin in SMALP, 9 μM protein, 18 μM liposomes, and 0.325 U agarose coupled trypsin were mixed in a final volume of 163 μL and incubated shaking at 150 rpm for 1 h at room temperature. After 1 h, SMA200 (Cube Biotech) was added in a ratio of 1.5:1 (w/w) polymer to lipid, and the mixture was incubated another hour shaking at 150 rpm and room temperature. Subsequently, trypsin was removed by centrifugation at 500*g* for 10 min at room temperature. The supernatant was used for the cryo-EM grid preparation.

### Preparation of Aerolysin in A8-35 and LMNG-CHS

For the preparation of aerolysin in LMNG and A8-35, aerolysin (20 μM) was mixed with 0.05% LMNG-CHS or A8-35 in a final volume of 400 μL. 4 μL of agarose-coupled or soluble trypsin (1 mg/mL) was added, and proteolytic activation was performed at 24 °C on a rotary shaker for 1 h. The sample was dialyzed with a cutoff of 3.5 kDa at room temperature for 2 h against 20 mM HEPES and 100 mM NaCl pH 7 prior to vitrification and cryo-EM. In the case of aerolysin Y221G, the sample was activated at 8 μM and concentrated to 20–30 μM after dialysis.

### Cryo-EM Grid Preparation

For the SMALP data sets, cryo-samples were prepared using a Vitrobot Mark IV (Thermo Fisher Scientific). Grids were frozen at 8 °C with a humidity of 95%. Quantifoil R1.2/1.3 on Cu 300 mesh grids coated with 5 nm carbon were glow discharged for 10 s at 10 mA using the GloQube Plus Glow Discharge System from Quorum. 4 μL of the sample was applied to the grid, and after 1 min incubation, the grids were blotted for 4 s and quickly plunged into liquid ethane precooled by liquid nitrogen. After vitrification, the grids were stored in liquid nitrogen until data collection. For the amphipol and LMNG data sets, vitrification was performed at 100% humidity using Quantifoil R1.2/1.3 and R 2/1 with 2 nm carbon. Grids were glow discharged on a Baltzer CT010 instrument for 5 s at 10 mA.

### Data Collection

The data sets for aerolysin in SMALP were collected at the Dubochet Center for Imaging (Lausanne, CH) using the 300 kV TFS Titan Krios G4 equipped with a Cold-FEG and Falcon 4 detection. The data sets were collected in the electron-counting mode (EER). The Falcon IV gain references were measured before starting data collection. The data collection was performed using the TFS EPU software packages. Movies were recorded at a nominal magnification of 96,000×, corresponding to 0.83 Å/pixel with defocus values ranging from 0.8 to 1.7. The exposure dose was set to 50 e/Å^2^. The amphipol, LMNG, and liposome data sets were collected at the Dubochet Center for Imaging (Bern, CH) on a 300 kV TFS Titan Krios G4 equipped with a Cold-FEG, Falcon 4i, and Selectris energy filter with a slit of 20 eV and an average exposure dose set to 40 e/Å^2^. The data were recorded and are summarized in the supplements (Supporting Information Table S4).

### Data Processing for Aerolysin

All data sets of aerolysin in SMALP were processed in cryoSPARC.^35^ The motion correction was performed on raw stacks without binning using the cryoSPARC Patch motion correction implementation. For all data sets, the initial CTF parameters were estimated using the Patch CTF estimation followed by particle picking using the blob picking tool. After several rounds of 2D classification, an Ab initio model was built that was used for template picking. Particles obtained from template picking were again cleaned by several rounds of 2D classification and by the construction of multiple Ab initio models. The selected particles were used for Ab initio reconstruction followed by homogeneous refinement applying C7 symmetry. The reported resolutions are based on the gold-standard Fourier shell correlation (FSC) = 0.143 criteria,^36^ and local-resolution variations were estimated using CryoSPARC. Amphipol and LMNG data sets were processed in Relion.^37^ In brief, the EER data set (40 e/Å^2^) was converted to 40 frames tifs using relion_convert_to_tiff. Motion correction^38^ and CTF-estimation^39^ were performed followed by particle picking, 2D classification, and refinement.^40^ The best particles were polished and CTF refined according to Relion protocols.^41−43^ The reported resolutions are based on the gold-standard FSC = 0.143 criterion.^36^ A schematic protocol of the processing for each structure can be found in the Supporting Information (Figures S1–S7). The aerolysin in liposome data set was processed in cryoSPARC. First 23,653 particles were selected manually, and after cleaning 13,057 particles were used for 2D reconstruction.

### Cryogenic Electron Tomography

For the incorporation of aerolysin WT in liposomes, 9 μM protein, 18 μM liposomes, and 0.325 U agarose coupled trypsin were mixed in a final volume of 163 μL and incubated shaking at 150 rpm for 2 h at room temperature. The agarose was removed by centrifugation at 500*g* for 10 min, and the supernatant was mixed with 10 nm gold particles and plunge frozen using a Vitrobot Mark IV (Thermo Fisher Scientific). Lacy grids were glow discharged for 10 s at 10 mA. Grids were frozen at 8 °C with a humidity of 95%. Tilt series were acquired every 3 degrees with a total dose of 100 e/A^2^ on the TFS Titan Krios G4 with Falcon IVi detector. The data were processed using IMOD.^44^

### Model Building

For model building of the aerolysin in SMALP data sets, the PDB structure of aerolysin (PDB ID: 5JZT) was fit into the cryo-EM map using Phenix^45^ and was manually adjusted using Coot^46^ for the K238A mutant. For the other structures, the model of the WT aerolysin in amphipol was used as an initial model and fit similarly. The final model was generated by iterating between manual model building in Coot and relaxed refinement in Rosetta^47^ and Phenix. In the wt structure in SMALP, amino acids 15–24 were not built because the density in this area was not well refined. Similarly, residues 10–26 and 246–251 in the K238A mutant in SMALP and residues 13–24 in the K238A–K244A mutant were not built. MolProbidity^48^ and EMRinger^49^ were used to validate the final model. The structure was analyzed using UCSF Chimera and UCSF ChimeraX.^50,51^ The dimensions of the pores were calculated using the software HOLE.^29^ Figures were prepared in a UCSF ChimeraX.

Model building of the amphipol structure was performed using ModelAngelo^52^ followed by Phenix Dock and Rebuild. The best chain was built by iterating between Coot and Phenix. For the flexible loops in domain 1 for which the density does not allow accurate modeling, the X-ray structure of the aerolysin monomer was used as a guide since the domain was shown to be stable.^32^

### MD Simulation Setup

The all-atom molecular dynamics simulations were prepared with the CHARMM-GUI^53^ Website. We set standard protonation states at pH 7 assisted by manual intervention for key residues whose protonation states were inferred from the high-resolution structures: H132, H186, and H332 were doubly protonated, H107 and H341 were modeled with a proton on the nitrogen ε, H121 protonated on the nitrogen δ, and E237 was protonated to establish the H-bonding pattern, as shown in Figure 1f, except when explained otherwise (i.e., keeping E237 in the charged form to test its effect, Supporting Information Figure S9). Using PPM2.0 through the CHARMM-GUI interface, the proteins were embedded in the corresponding membranes, i.e., either DPhPC or DOPC/DOPE (2:1) as pertinent in each case. The systems were further prepared (solvated and neutralized) with standard CHARMM-GUI procedures and parameters, either in 1 M KCl for current measurements under voltage or 0.15 M KCl when testing protein behavior in membranes without voltage. The systems were parametrized in CHARMM-GUI using CHARMM36m^54^ for the protein components and the corresponding version of TIP3P water, plus the standard CHARMM parameters available for lipids. After standard CHARMM-GUI-provided minimization and equilibration procedures with the parameters and restrain strengths described in Supporting Information Table S5 and using Gromacs 2022 or 2023, we ran the production simulations using the same MD engines and standard production files from CHARMM-GUI, i.e., semi-isotropic pressure coupling to 1 atm, a temperature of 298 K, 2 fs integration steps, LINCS-based restraints on hydrogens, and PME electrostatics with 12 Å cutoff. Details specific to each simulation are given in Supporting Information Table S5. RMSD and RMSF plots are reported for all MD simulations (Supporting Information Figure S13).

### Water Accessibility Radius^55^

For the calculation of the hydrodynamic radius, a density map of water and the pore was computed over the box. Based on these density map, with a radius step d*r*, the average density of water and pore in a ring centered on the pore axis with a radius *r*, i.e., the density between *r* and *r* + d*r* was computed. Once the ratio between the density of water and the density of pore reached a threshold value, the last *r* value was retrieved and considered as the hydrodynamic radius. This was then repeated over the pore’s axis to obtain a radius value at each grid point on the pore’s axis.

### Ionic Current Calculation

The ionic currents were calculated according to the method developed in ref (55). Briefly, the current at time t is computed with the equation hereunder and then averaged over the trajectory.
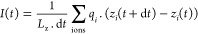
where *I*(*t*) is the current, *L*_z_ is the box size on the pore’s axis, d*t* is the time step between two frames, *q*_*i*_ is the charge of an ion, and *z*_*i*_ is its position on the pore axis.

The bulk conductivity in water boxes at 1 M KCl is 12.0 mS/cm. This result aligns well with the findings from previous studies^55^ where the estimated conductivity ranged between 12 and 13 mS/cm. Furthermore, it is also in good agreement with the experimental conductivity measured to be 11.3 ± 0.1 mS/cm at 25 °C, with a deviation of only 6.2%. Since the estimated error relative to the experimental values is limited, we did not apply any scaling factor to our current calculations.

## Supporting Information Available

Analysis of MD simulations for different aerolysin structures, detailed comparison of the polar interactions stabilizing aerolysin, cryoEM data collection, and refining process for all aerolysin models (PDF)
3D variability analysis performed in CryoSparc on Aerolysin WT in SMALP (MOV)
Orientation of aerolysin in the membrane, additionally confirmed by cryogenic electron tomography. The positions of a few pores on the liposome are highlighted with a red arrow (MP4)

## Supplementary Material

ja4c14288_si_001.pdf

ja4c14288_si_002.mov
