# Supplementary material for: Aerolysin Nanopore Structures Revealed at High Resolution in a Lipid Environment
Source: J Am Chem Soc. 2025 Feb 3;147(6):4984–92. doi: 10.1021/jacs.4c14288 (PMC11826888; doi:10.1021/jacs.4c14288)
Supplement: Supplementary file 1 — ja4c14288_si_001.pdf [file ja4c14288_si_001.pdf]

Supplementary Information

Aerolysin nanopore structure revealed at high resolution in lipid environment

Jana S. Anton[1#], Ioan Iacovache[2#], Juan F. Bada Juarez[1], Luciano A. Abriata[1], Louis W. Perrin[3], Chan Cao[3], Maria J. Marcaida[1], Benoît Zuber[2*], Matteo Dal Peraro[1*]

[1] Institute of Bioengineering, School of Life Sciences, École Polytechnique Fédérale de Lausanne (EPFL), 1015 Lausanne, Switzerland
[2] Institute of Anatomy, University of Bern, Baltzerstrasse 2, 3012 Bern, Switzerland
[3] Department of Inorganic and Analytical Chemistry, Chemistry and Biochemistry, University of Geneva, 1211 Geneva, Switzerland

[#] These authors contributed equally
* Corresponding authors: M.D.P. matteo.dalperaro@epfl.ch; B.Z. benoit.zuber@unibe.ch

Supplementary Table 1: Overview of the overall Cα RMSD (in Å) of the aerolysin structures calculated with ChimeraX.

Structure	WT amphipol	WT SMALP	K238A SMALP	K238A/K244A SMALP	5JZT
**WT amphipol**	0	0.816	0.92	1.065	3.15
**WT SMALP**	0.815	0	0.476	0.642	3.07
**K238A SMALP**	0.92	0.476	0	0.438	2.95
**K238A/K244A SMALP**	1.065	0.642	0.438	0	2.992
**5JZT**	3.15	3.07	2.95	2.992	0

Supplementary Table 2 : Overview of the Cα RMSD (in Å) of the aerolysin barrel (residue 215-285) between the different aerolysin structures. The RMSD was calculated using ChimeraX.

Structure	WT amphipol	WT SMALP	K238A SMALP	K238A/K244A SMALP	5JZT
**WT amphipol**	0	0.74	0.85	0.727	2.06
**WT SMALP**	0.74	0	0.54	0.247	2.46
**K238A SMALP**	0.85	0.54	0	0.535	1.81
**K238A/K244A SMALP**	0.727	0.247	0.535	0	2.44
**5JZT**	2.06	2.46	1.81	2.44	0

Supplementary Table 3 : Overview of the H-bonds for aerolysin wt in amphipol and SMALP and for the Y221G pre-pore mutant. Interactions are only shown for chain A /X, where X represents the amino acid from protomer X. Analysis was performed using ChimeraX.

WT amphipol			WT SMALP			Y221G mutant		
**Donor**	**Acceptor**	**Distance (Å)**	**Donor**	**Acceptor**	**Distance (Å)**	**Donor**	**Acceptor**	**Distance (Å)**
/A TYR 5	/A TYR 76	3.2	A TYR 5	/A TYR 76	3.2	/A GLN 8	/A ASP 7	2.8
/A GLN 8	/A TYR 76	3.5	/A GLN 8	/A ASP 7	3.0	/A ARG 10	/A ASP 7	2.4
/A GLN 8	/A VAL 81	2.9	/A ASN 27	/A GLU 30	3.0	/A ARG 10	/A LEU 9	2.7
/A ARG 10	/A ASP 7	2.3	/A ARG 28	/G MET 140	3.0	/A ARG 10	/A ASP 7	3.0
/A ARG 10	/A LEU 9	2.5	/A ARG 28	/G ASP 139	2.6	/A ASN 27	/A GLU 30	3.4
/A ARG 10	/A ASP 7	3.0	/A ARG 28	/G ASP 141	3.3	/A ARG 28	/B MET 140	2.7
/A ARG 24	/A PRO 25	3.3	/A GLU 29	/A GLU 29	2.6	/A ARG 28	/B ASP 139	2.4
/A ARG 28	/B MET 140	3.5	/A GLN 32	/G VAL 142	3.1	/A ARG 28	/B MET 140	3.3
/A ARG 28	/B MET 140	3.5	/A SER 33	/A GLU 30	3.0	/A ARG 28	/B ASP 141	2.9
/A GLU 30	/A ASN 27	2.8	/A LYS 35	/G ASP 156	3.4	/A GLU 30	/A ASN 27	2.5
/A GLN 32	/B VAL 142	3.4	/A GLY 43	/A GLN 46	2.7	/A GLN 32	/B VAL 142	3.1
/A SER 33	/A GLU 30	2.6	/A SER 48	/A ASN 72	2.6	/A LYS 35	/A GLY 63	2.9
/A LYS 35	/A GLY 63	3.4	/A TYR 61	/A TRP 45	2.8	/A GLY 43	/A GLN 46	2.5
/A GLY 43	/A GLN 46	2.6	/A ASN 72	/A ILE 47	3.1	/A GLU 84	/A GLU 84	2.7
/A GLN 46	/A SER 48	3.4	/A ASN 72	/A SER 71	3.2	/A VAL 99	/A ASP 97	2.7
/A ASN 72	/A ILE 47	3.0	/A CYS 75	/A THR 73	3.6	/A GLN 102	/A TYR 177	3.1
/A THR 78	/A LYS 22	2.7	/A GLU 98	/A GLU 98	2.8	/A HIS 107	/A TRP 103	3.0
/A ILE 85	/A GLU 84	3.1	/A ASP 100	/A ASP 97	3.0	/A ASN 111	/A ASP 108	3.1
/A GLU 98	/A GLU 98	3.0	/A ARG 104	/B GLU 2	3.0	/A ASN 111	/A ASP 108	2.7
/A VAL 99	/A ASP 97	3.4	/A ASN 111	/A ASP 108	3.1	/A TRP 127	/A TYR 162	3.0
/A ASP 100	/A ASP 97	2.8	/A ASN 111	/A ASP 108	3.0	/A ASN 131	/A ASN 155	3.4
/A GLN 102	/A TYR 177	3.4	/A LYS 114	/A VAL 136	2.7	/A ASN 131	/A ASP 156	3.0
/A ARG 104	/G GLU 2	3.4	/A LYS 114	/A GLU 138	2.9	/A HIS 132	/A ASP 139	2.9
/A HIS 107	/A TRP 103	2.9	/A THR 116	/A PHE 112	2.8	/A HIS 132	/A ASP 139	2.8
/A ASN 111	/A ASP 108	3.4	/A SER 117	/A ILE 113	2.7	/A HIS 132	/G GLU 64	0.0
/A ASN 111	/A ASP 108	3.4	/A TRP 127	/A TYR 162	3.1	/A SER 133	/A GLU 138	2.7
/A ASN 111	/A ASP 108	3.0	/A ASN 131	/A ASN 155	2.9	/A TYR 135	/A SER 133	3.2
/A LYS 114	/A VAL 136	2.5	/A ASN 131	/A GLY 157	3.1	/A VAL 142	/G GLN 32	3.1
/A THR 116	/A PHE 112	3.1	/A HIS 132	/A ASP 139	2.8	/A ARG 144	/G GLU 367	3.0

/A SER 117	/A ILE 113	2.8	/A HIS 132	/A ASP 139	3.0	/A ARG 144	/A GLY 148	3.5
/A TYR 125	/A LEU 318	3.4	/A HIS 132	/B GLU 64	3.1	/A TRP 149	/A GLN 102	3.1
/A ASN 131	/A ASN 155	3.4	/A GLN 134	/A GLN 134	2.6	/A ARG 163	/A ASP 166	2.9
/A HIS 132	/A ASP 139	3.3	/A TYR 135	/A SER 133	3.1	/A ARG 163	/A ARG 336	3.3
/A HIS 132	/A ASP 139	2.7	/A VAL 142	/B GLN 32	2.9	/A ARG 163	/A ASP 166	3.2
/A HIS 132	/G GLU 64	2.8	/A THR 143	/A ARG 144	3.6	/A LYS 167	/A ASN 131	2.7
/A VAL 142	/G GLN 32	3.2	/A ARG 144	/B GLU 367	3.1	/A LYS 167	/A ASN 155	2.8
/A THR 143	/A VAL 150	3.5	/A ARG 144	/A GLN 102	3.2	/A ASN 174	/A ASP 315	3.0
/A ARG 144	/G GLU 367	3.2	/A TRP 149	/A GLN 102	2.9	/A TYR 177	/A GLU 98	2.9
/A ARG 144	/A GLN 102	3.3	/A ASN 154	/A ASP 141	2.9	/A ASP 182	/A ASP 180	3.2
/A TRP 149	/A GLN 102	3.1	/A ASN 154	/A ASP 156	2.9	/A HIS 186	/A GLY 187	2.8
/A ASN 154	/A ASP 141	2.6	/A ASP 156	/A ASN 154	3.0	/A SER 192	/A ASP 193	3.5
/A ASN 154	/A ASP 156	3.3	/A ARG 163	/A ASP 166	3.0	/A ASN 206	/A THR 210	2.9
/A ASP 156	/A ASN 154	3.3	/A ARG 163	/A ASP 166	3.1	/A ASN 206	/A VAL 285	2.8
/A LYS 167	/A GLY 129	3.0	/A LYS 167	/A GLY 129	2.9	/A SER 208	/A ASN 206	2.9
/A LYS 167	/A ASN 131	3.1	/A LYS 167	/A ASN 131	2.9	/A SER 208	/A ASN 206	2.8
/A THR 168	/A ALA 126	2.9	/A THR 168	/A ALA 126	2.6	/A THR 210	/A SER 208	3.2
/A ASN 174	/A ASP 315	3.5	/A ASN 174	/A ASP 315	2.9	/A GLN 212	/A SER 208	3.3
/A SER 183	/A ASP 180	3.0	/A SER 183	/A ASP 180	2.9	/A GLN 212	/G THR 284	3.4
/A SER 183	/A LYS 309	3.4	/A SER 183	/A LYS 309	3.5	/A TYR 215	/A VAL 205	2.6
/A LYS 185	/A GLU 307	2.8	/A HIS 186	/A GLY 187	3.3	/A THR 225	/A THR 273	3.0
/A HIS 186	/A GLY 187	2.9	/A HIS 186	/G GLU 415	2.9	/A LYS 229	/A TRP 265	2.6
/A HIS 186	/B GLU 415	2.9	/A THR 190	/A SER 303	3.5	/A LYS 229	/A GLN 268	2.5
/A HIS 186	/B GLU 415	3.1	/A ARG 194	/A GLN 191	2.8	/A ASN 231	/A ALA 266	3.5
/A THR 190	/A SER 303	3.4	/A GLN 195	/A ASP 193	3.1	/A ASN 231	/A ASN 269	2.8
/A SER 192	/A ASP 193	3.0	/A LYS 198	/B SER 408	3.3	/A LYS 242	/A ASN 178	3.2
/A ARG 194	/A GLN 191	2.6	/A THR 199	/B GLN 409	2.8	/A TRP 247	/A TYR 304	2.8
/A ARG 194	/A GLN 191	3.1	/A THR 199	/B GLN 409	3.3	/A ALA 260	/A GLN 268	3.0
/A THR 199	/G GLN 409	3.1	/A ASN 206	/A THR 210	3.0	/A TRP 265	/A TYR 306	3.1
/A THR 199	/A ILE 295	3.0	/A ASN 206	/A VAL 285	3.1	/A SER 267	/A SER 264	2.6
/A ASN 206	/A THR 210	2.8	/A SER 208	/A ASN 206	2.9	/A SER 267	/A SER 264	3.3
/A ASN 206	/A VAL 285	3.1	/A SER 208	/A ASN 206	2.8	/A GLN 268	/A ALA 260	2.9

/A ASN 206	/A PRO 286	3.1	/A THR 210	/A SER 208	3.3	/A GLN 268	/A GLN 263	3.3
/A SER 208	/A ASN 206	3.0	/A THR 210	/A PRO 211	3.4	/A ASN 269	/A ALA 266	2.7
/A SER 208	/A ASN 206	2.5	/A GLN 212	/A SER 208	3.3	/A GLN 279	/A GLU 296	3.0
/A SER 208	/A THR 210	3.2	/A GLN 212	/B THR 284	3.3	/A ARG 282	/A ASP 216	2.5
/A THR 210	/A PRO 211	3.5	/A TYR 215	/A VAL 205	2.8	/A THR 284	/B GLN 212	3.1
/A GLN 212	/A SER 208	3.0	/A THR 218	/A ASP 216	2.8	/A ARG 288	/A ASP 207	3.1
/A GLN 212	/G THR 284	3.3	/A THR 218	/A SER 280	2.7	/A ARG 288	/A ASP 209	3.5
/A SER 213	/A THR 284	3.2	/A ARG 220	/A ASP 222	3.2	/A ARG 288	/A ASP 209	3.1
/A TYR 215	/A VAL 205	2.9	/A SER 228	/B GLN 268	2.7	/A SER 289	/A PRO 286	2.6
/A THR 218	/A ASP 216	2.9	/A LYS 238	/A GLU 258	2.8	/A ASP 301	/A SER 192	2.8
/A THR 218	/A ASP 216	3.0	/A LYS 242	/A GLU 254	2.9	/A TYR 306	/A THR 230	2.9
/A THR 218	/A SER 280	2.3	/A ASN 243	/B GLU 252	2.9	/A LYS 309	/A SER 183	2.9
/A ARG 220	/A ASP 222	3.2	/A ASN 243	/B THR 253	2.7	/A LYS 309	/A GLU 307	3.0
/A ARG 220	/A ASP 222	3.0	/A THR 253	/A GLY 251	3.4	/A ARG 323	/A HIS 121	3.0
/A ARG 220	/A ASP 222	2.9	/A SER 264	/A TYR 233	3.5	/A ARG 323	/A TYR 125	3.3
/A SER 228	/A ASN 269	3.4	/A GLN 268	/G ASN 226	3.0	/A ASN 327	/A ARG 323	3.2
/A SER 236	/A ALA 261	3.3	/A SER 276	/A ASP 222	2.7	/A ASN 327	/A PRO 333	2.8
/A LYS 238	/G GLU 258	3.4	/A GLN 279	/A GLU 296	3.4	/A TRP 329	/A ASN 327	3.2
/A LYS 242	/A SER 256	2.7	/A SER 280	/A THR 218	2.7	/A HIS 332	/A ASN 327	3.0
/A LYS 244	/A GLU 254	3.0	/A ARG 282	/A ASP 216	2.7	/A ASN 340	/A SER 319	2.8
/A LYS 244	/A GLU 254	3.3	/A ARG 282	/A ASP 216	3.1	/A LYS 351	/A TYR 357	3.5
/A THR 253	/A GLY 251	3.2	/A THR 284	/G GLN 212	2.9	/A SER 353	/A ASP 350	3.0
/A THR 253	/B ASN 243	3.4	/A ARG 288	/A ASP 209	3.0	/A ARG 356	/A ILE 85	3.4
/A GLN 263	/A GLU 237	3.2	/A ARG 288	/A ASP 207	3.0	/A ARG 356	/A ILE 85	3.5
/A GLN 268	/A SER 228	2.5	/A ARG 288	/A ASP 209	3.0	/A TYR 357	/A SER 354	2.9
/A GLN 268	/B ASN 226	3.1	/A SER 289	/A PRO 286	2.7	/A GLN 358	/A LYS 351	3.1
/A SER 276	/A ASP 222	2.4	/A LYS 290	/C PRO 181	3.0	/A GLN 358	/A ALA 352	2.9
/A GLN 279	/A GLU 296	3.3	/A LYS 294	/A GLU 296	2.9	/A LYS 361	/B ASP 97	3.2
/A SER 280	/A ASP 216	3.3	/A TYR 298	/A GLU 296	2.9	/A ARG 362	/A TRP 359	2.9
/A SER 280	/A THR 218	2.3	/A SER 303	/A PHE 404	3.3	/A TRP 373	/A GLN 358	2.9
/A ARG 282	/A ASP 216	3.1	/A SER 303	/B TYR 348	2.8	/A ASN 374	/A ARG 362	2.9
/A THR 284	/B GLN 212	3.1	/A TYR 304	/G GLU 296	2.8	/A TRP 375	/A ASP 372	3.0

/A THR 284	/A SER 213	3.2	/A TYR 306	/A GLU 98	2.8	/A ASN 380	/A THR 376	3.4
/A SER 289	/A PRO 286	2.3	/A LYS 309	/A SER 183	3.1	/A GLN 386	/A GLU 84	3.4
/A SER 289	/A ALA 287	3.4	/A SER 313	/A ALA 176	3.5	/A ASN 388	/A PRO 115	3.0
/A LYS 290	/F PRO 181	2.5	/A THR 317	/A THR 342	2.7	/A ARG 394	/A SER 89	3.0
/A LYS 299	/A SER 408	2.8	/A SER 319	/A ASN 340	2.7	/A ARG 397	/A ASP 92	2.9
/A LYS 299	/A GLN 409	3.4	/A ASN 327	/A ARG 323	3.4	/A ARG 397	/A ASP 92	2.9
/A ASP 301	/A ASP 193	3.0	/A ASN 327	/A PRO 333	3.2	/A THR 401	/A ASP 95	3.2
/A SER 303	/A SER 405	2.9	/A TRP 329	/A ASN 327	3.0	/A THR 401	/A ASP 95	2.9
/A SER 303	/G TYR 348	3.1	/A HIS 332	/A ASN 327	3.0	/A ASP 403	/A THR 230	3.0
/A TYR 304	/B GLU 296	3.1	/A THR 342	/A THR 317	2.7	/A ASN 413	/A GLU 296	3.0
/A TYR 306	/A GLU 98	2.9	/A LYS 349	/G SER 192	3.0	/A ASN 413	/A GLU 415	2.8
/A LYS 309	/A SER 183	2.9	/A LYS 349	/G ASP 301	3.0			
/A SER 313	/A ALA 176	3.4	/A LYS 351	/A ASP 350	2.6			
/A THR 317	/A THR 342	3.1	/A ARG 356	/A ILE 85	3.2			
/A SER 319	/A ASN 340	2.3	/A TYR 357	/A SER 354	3.2			
/A ARG 323	/A HIS 121	3.3	/A TYR 357	/A GLU 367	2.8			
/A ARG 323	/A TYR 125	2.5	/A GLN 358	/A LYS 351	3.1			
/A ARG 323	/A HIS 121	3.3	/A GLN 358	/A ALA 352	3.1			
/A ASN 327	/A ARG 323	3.2	/A TRP 359	/A GLY 83	2.8			
/A ASN 327	/A PRO 333	3.0	/A LYS 361	/G ASP 97	2.8			
/A TRP 329	/A ASN 327	3.2	/A ARG 362	/A TRP 359	3.2			
/A HIS 332	/A ASN 327	2.9	/A ARG 362	/A THR 82	2.8			
/A ASN 338	/A GLY 320	3.0	/A TYR 363	/G ASP 100	2.6			
/A THR 342	/A THR 317	3.1	/A TRP 373	/A GLN 358	3.2			
/A LYS 351	/A TYR 357	2.7	/A ASN 374	/A ASP 372	2.8			
/A SER 353	/A ASP 350	2.9	/A ASN 374	/A ARG 362	3.1			
/A SER 353	/A ASP 350	3.4	/A TRP 375	/A ASP 372	3.0			
/A SER 353	/A ASP 350	3.1	/A THR 376	/A TRP 373	2.9			
/A ARG 356	/A ILE 85	3.4	/A ASN 380	/A THR 376	3.2			
/A TYR 357	/A GLU 367	2.9	/A THR 384	/A ASN 380	2.8			
/A GLN 358	/A LYS 351	3.1	/A GLN 386	/A GLU 84	2.9			
/A GLN 358	/A ALA 352	3.2	/A ARG 394	/A SER 89	2.8			

/A TRP 359	/A GLY 83	2.6	/A ARG 397	/A ASP 311	3.0			
/A LYS 361	/B ASP 97	3.0	/A ARG 397	/A ASP 180	3.4			
/A LYS 361	/B ASP 100	3.2	/A ARG 397	/A ASP 180	2.7			
/A ARG 362	/A TRP 359	2.4	/A GLY 399	/A ASP 95	2.9			
/A TYR 363	/B ASP 100	2.4	/A SER 405	/B LYS 349	2.8			
/A TRP 373	/A GLN 358	2.9	/A SER 408	/A ASP 301	3.0			
/A ASN 374	/A ARG 362	3.0	/A GLN 409	/A GLU 407	2.8			
/A TRP 375	/A ASP 372	3.4						
/A THR 376	/A TRP 373	2.7						
/A ASN 380	/A THR 376	3.2						
/A THR 384	/A ASN 380	3.5						
/A GLN 386	/A GLU 84	2.9						
/A ASN 388	/A PRO 115	3.0						
/A ARG 394	/A SER 89	2.5						
/A ARG 394	/A SER 89	3.2						
/A ARG 394	/A SER 89	3.1						
/A ARG 397	/A ASP 180	3.5						
/A ARG 397	/A ASP 180	3.5						
/A ARG 397	/A ASP 311	3.5						
/A ARG 397	/A ASP 180	2.8						
/A SER 405	/A SER 303	2.9						
/A SER 405	/G TYR 348	3.5						
/A SER 405	/G LYS 349	3.1						
/A SER 408	/A ASP 301	2.9						
/A GLN 409	/A GLU 407	2.7						

Supplementary Table 4 : CryoEM maps processing and model refinement statistics

Protein	WT SMALP	K238A SMALP	K238A K244A SMALP	WT LMNG	Aeroylsin Y221G	Aerolysin WT in Amphipol
**Access code**						
PDB	9FM6	9FNP	9FNQ		9FMX	9FML
EMDB	EMD-50549	EMD-50601	EMD-50602	EMD-50578	EMD-50576	EMD-50562
**Data collection and processing**						
Microscope	TFS Titan Krios G4	TFS Titan Krios G4	TFS Titan Krios G4	TFS Titan Krios G4	TFS Titan Krios G4	TFS Titan Krios G4
Detector	Falcon IV	Falcon IV	Falcon IV	Falcon IVi	Falcon IVi	Falcon IVi
Recording mode	electron-counting mode (EER)	electron-counting mode (EER)	electron-counting mode (EER)	electron-counting mode (EER)	electron-counting mode (EER)	electron-counting mode (EER)
Magnification	96000	96000	96000	165000	165000	130000
Voltage (kV)	300	300	300	300	300	300
Total dose (e^-/Å^2)	50	50	50	40	40	40
Nominal under focus range (µm)	0.8 - 1.7	0.8 - 1.7	0.8 - 1.7	0.8 - 2.5	0.8 - 2.5	0.8 - 2.5
Pixel size (Å)	0.83	0.83	0.83	0.73	0.73	0.94
Number of movie micrographs	10825	10216	8779	10596	34277	16526
Number of molecular projection images in map	89384	120835	139950	133420	329000	1070455
Symmetry	C7	C7	C7	C7	D7	C7
Map resolution (Å)	2.17	2.16	2.1	2.6	1.9	2.2
Map sharpening B-factor	-47.6	-55	-38.8	-65.8	-35.32	-43.55
**Model refinement and validation**						
Residues	2884	2793	2870		5936	2954
RMSD BondLength (4**σ**)	0.009	0.006	0.003		0.005	0.003
RMSD Bond Angles (4**σ**)	1.153	0.878	0.567		0.634	0.656
Ramachandran Outliers (%)	0	0	0		0	0.24
Ramachandran Allowed (%)	1.47	1.78	0.99		2.95	3.33

Ramachandran Favored (%)	98.53	98.22	99.01		97.05	96.43
Rotamer outliers (%)	0.29	1.19	1.16		1.13	1.69
Clash score	0.61	1.76	2.25		3.61	7.32
Molprobity score	0.7	0.99	1.05		1.36	1.81
EMRinger score	6.6	6.4	5.56		6.55	6.08

Supplementary Table 5: Parameters used for all-atom molecular dynamics simulations

System			Minimization	Equilibration	Production	Voltage (mV)
Membrane	Pore	Ions				
DOPC:DOPE (2:1)	WT SMALP with E237 either in the observed (neutral) protonation state, or charged	KCl (0.15M)	Restrains: LINCS algorithm on H-bonds<br><br>Lipid FC: 1000<br>Dihedral FC: 1000<br>Backbone:4000<br><br>Run until Fmax < 1000 with steep integration	6 classical CHARMM-GUI equilibration + Last step extended for 6ns.<br><br>Temperature: 298.15K	400ns<br><br>Temperature: 298.15K	0 (only membrane insertion and stability being tested)
DPhPC	K238A SMALP	KCl (1M)	same	6 classical CHARMM-GUI equilibration + Last step extended for 6ns.<br><br>Temperature: 298.15K	250ns<br><br>Temperature: 298.15K	-150<br>-100<br>-50<br>50<br>100<br>150<br>200

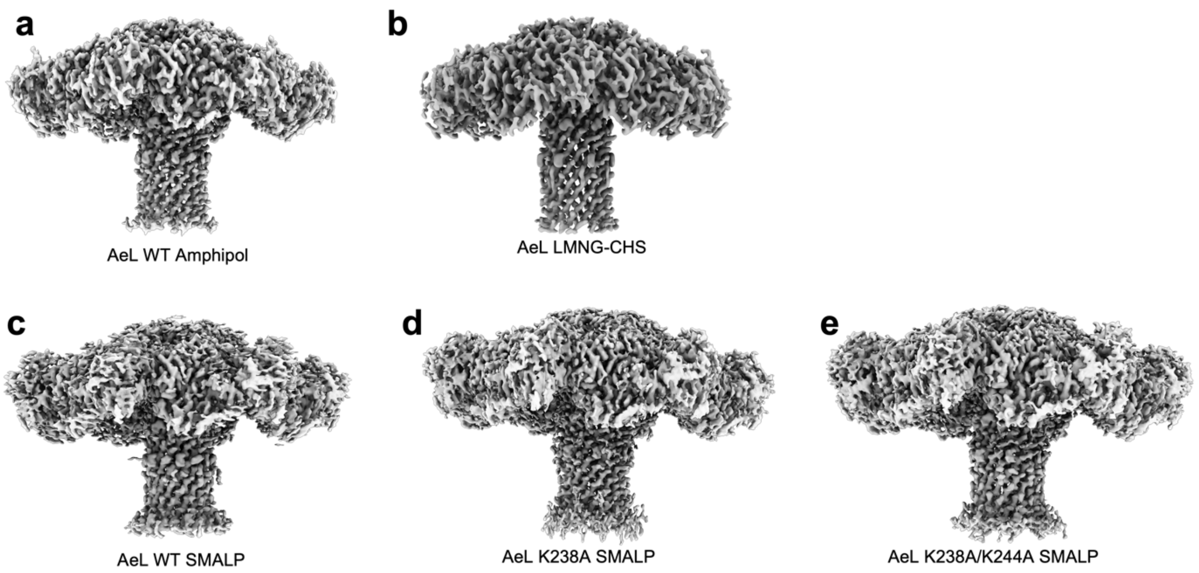

descriptions

Supplementary Figure 1 : Overview of the density maps generated by cryoEM.

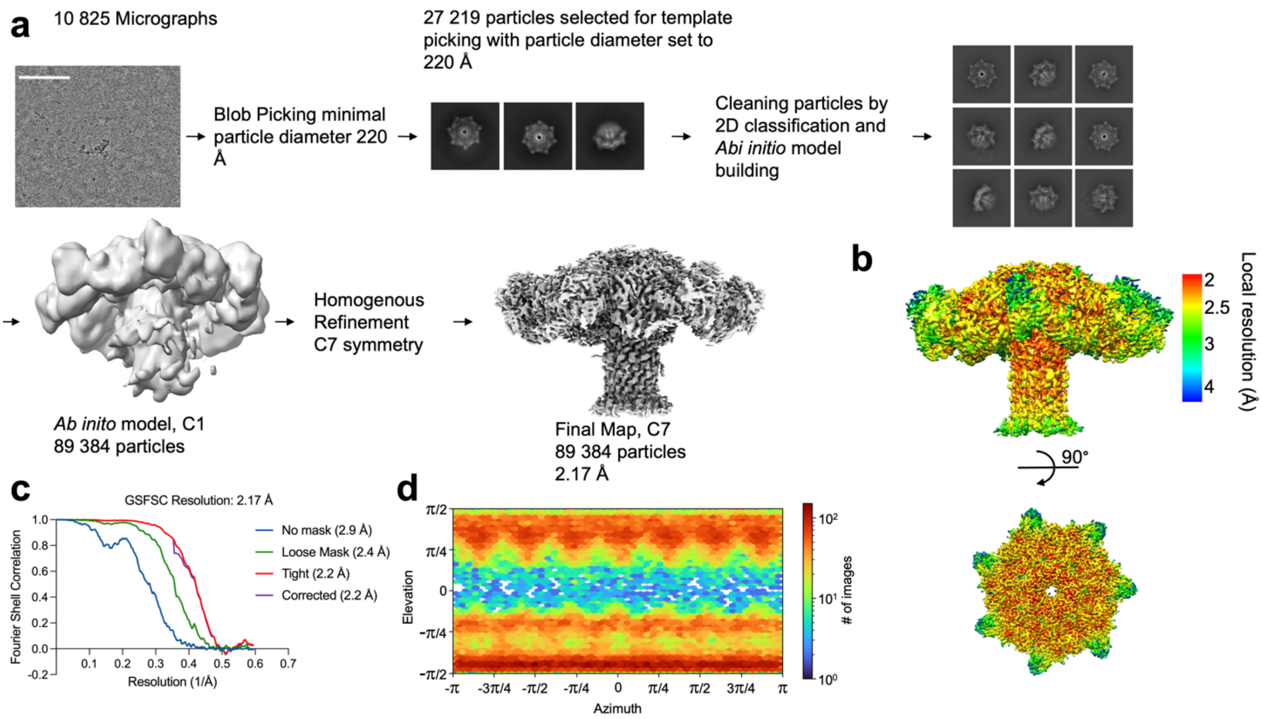

Supplementary Figure 2 : Cryo-EM processing of aerolysin WT in SMALPs. a Flow chart of the data processing in cryoSPARC. Scale bar represent 100 nm. **b** Final 3D reconstruction of aerolysin wild-type in SMALP colored by resolution. **c** Golden-standard Fourier shell correlation curves for the 3D reconstruction. **d** Angular distribution of the particles included in the final reconstruction.

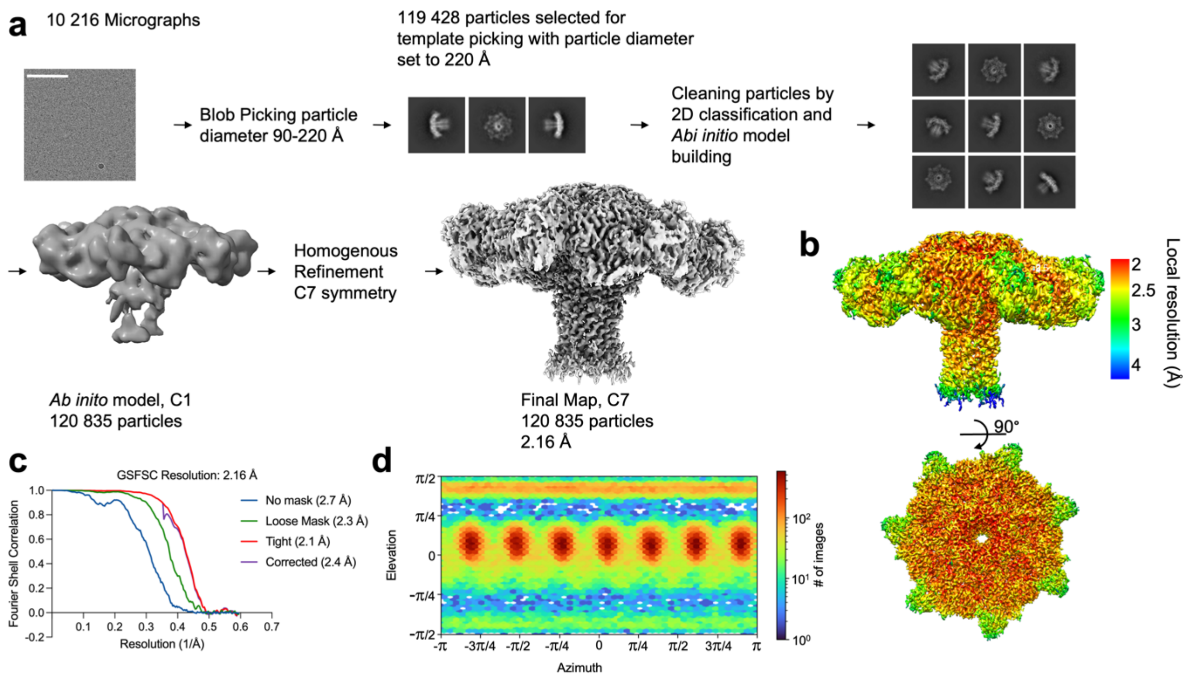

Supplementary Figure 3 : Cryo-EM processing of aerolysin mutant K238A in SMALPs. a Flow chart of the data processing in cryoSPARC. Scale bar represent 100 nm. b Final 3D reconstruction of aerolysin K238A in SMALP colored by resolution. c Golden-standard Fourier shell correlation curves for the 3D reconstruction. d Angular distribution of the particles included in the final reconstruction.

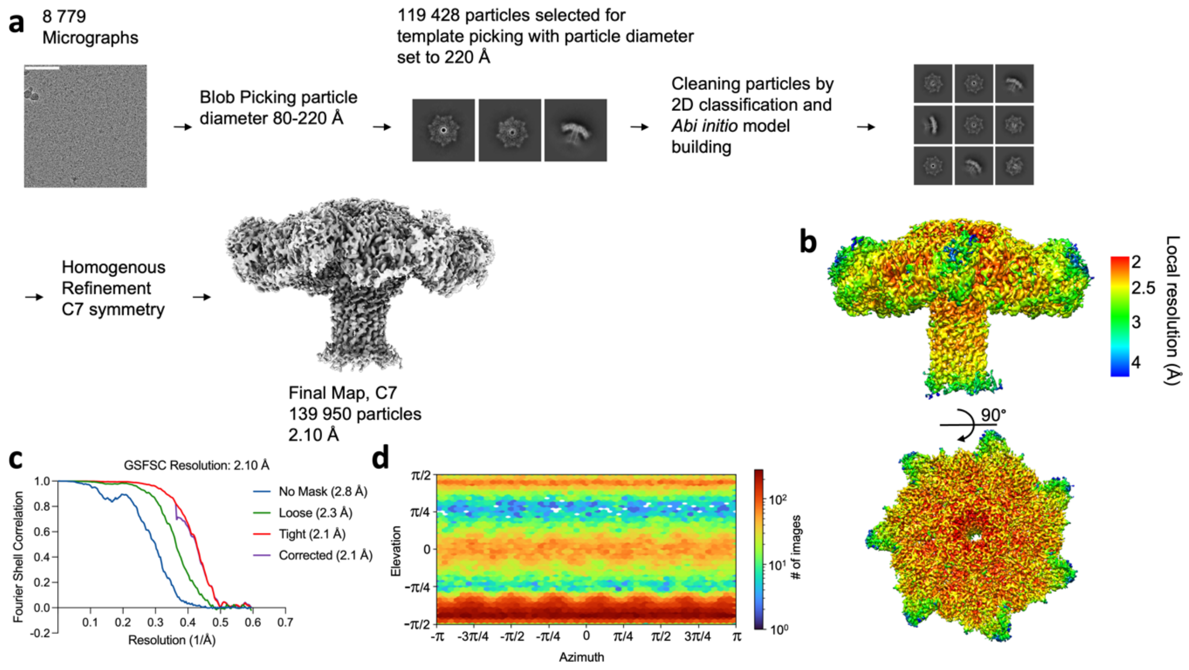

Supplementary Figure 4 : Cryo-EM processing of aerolysin mutant K238A/K244A in SMALPs. **a** Flow chart of the data processing in cryoSPARC. Scale bar represent 100 nm. **b** Final 3D reconstruction of aerolysin K238A/K244A in SMALP colored by resolution. **c** Golden-standard Fourier shell correlation curves for the 3D reconstruction. **d** Angular distribution of the particles included in the final reconstruction.

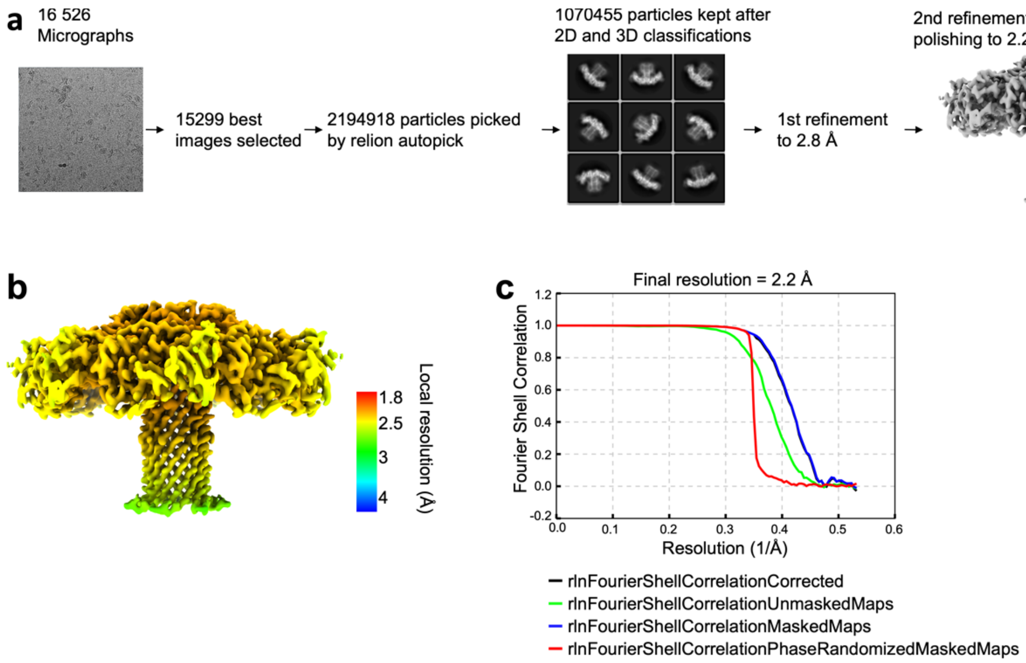

Supplementary Figure 5 : Cryo-EM processing of aerolysin WT in amphipol. **a** Flow chart of the data processing in Relion. Scale bar represent 100 nm**. b** Final map colored by resolution. **c** Golden-standard Fourier shell correlation curves for the 3D reconstruction.

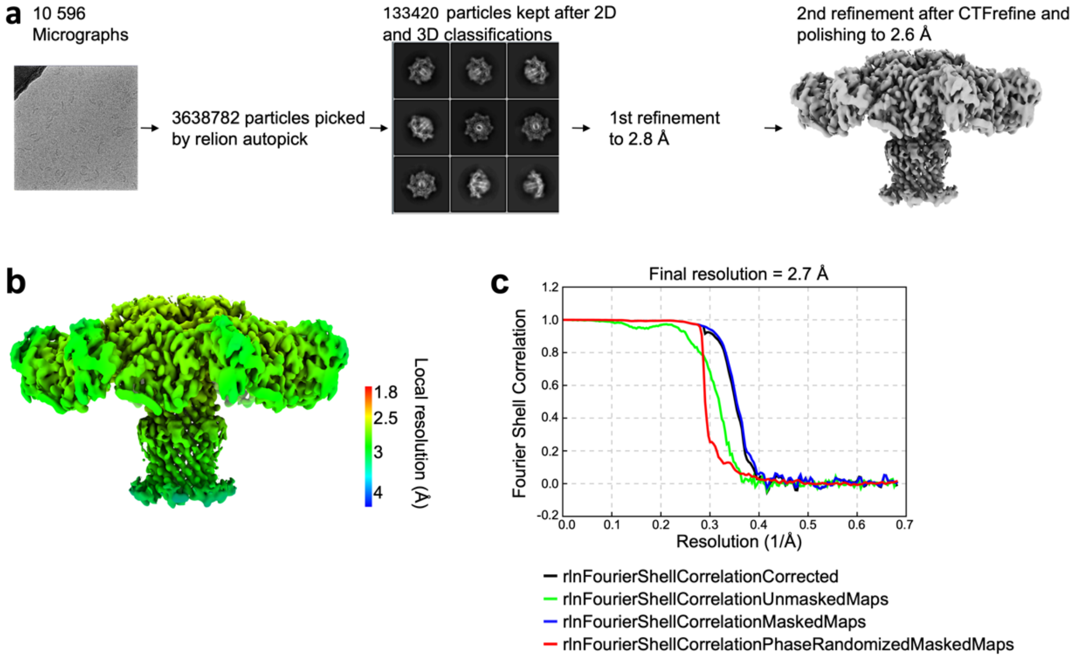

Supplementary Figure 6 : Cryo-EM processing of aerolysin WT in LMNG:CHS. **a** Flow chart of the data processing in Relion. Scale bar represent 100 nm. **b** Final map colored by resolution. **c** Golden-standard Fourier shell correlation curves for the 3D reconstruction.

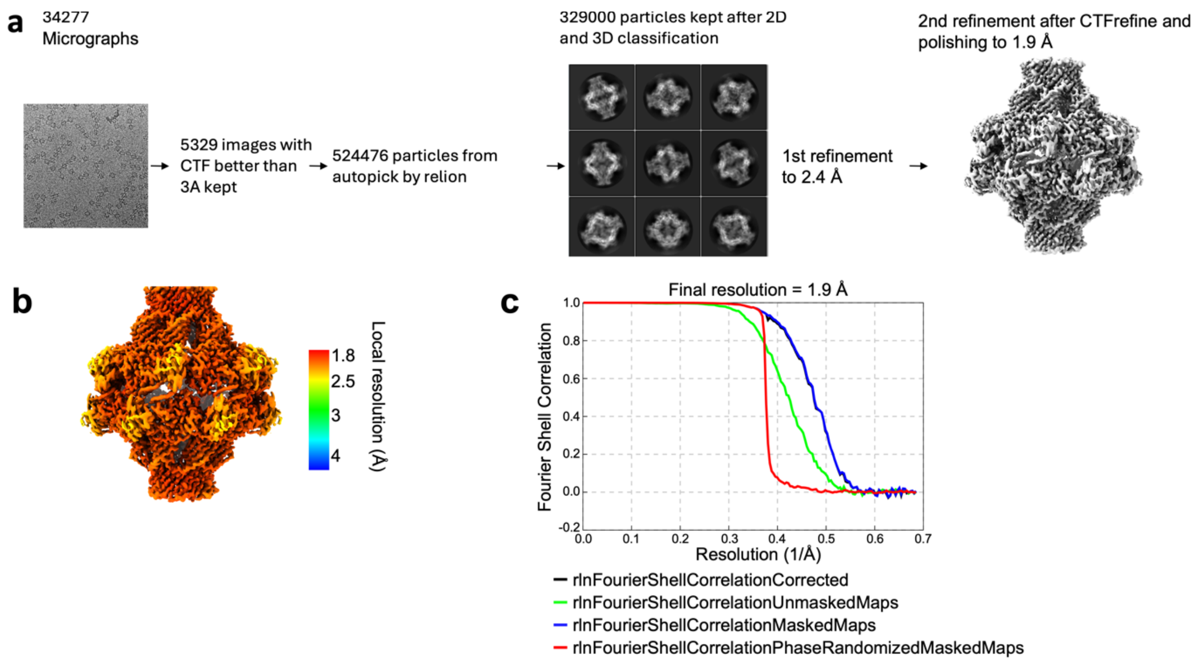

Supplementary Figure 7 : Cryo-EM processing of aerolysin mutant Y221G. **a** Flow chart of the data processing in Relion. Scale bar represent 100 nm. **b** Final map colored by resolution. **c** Golden-standard Fourier shell correlation curves for the 3D reconstruction.

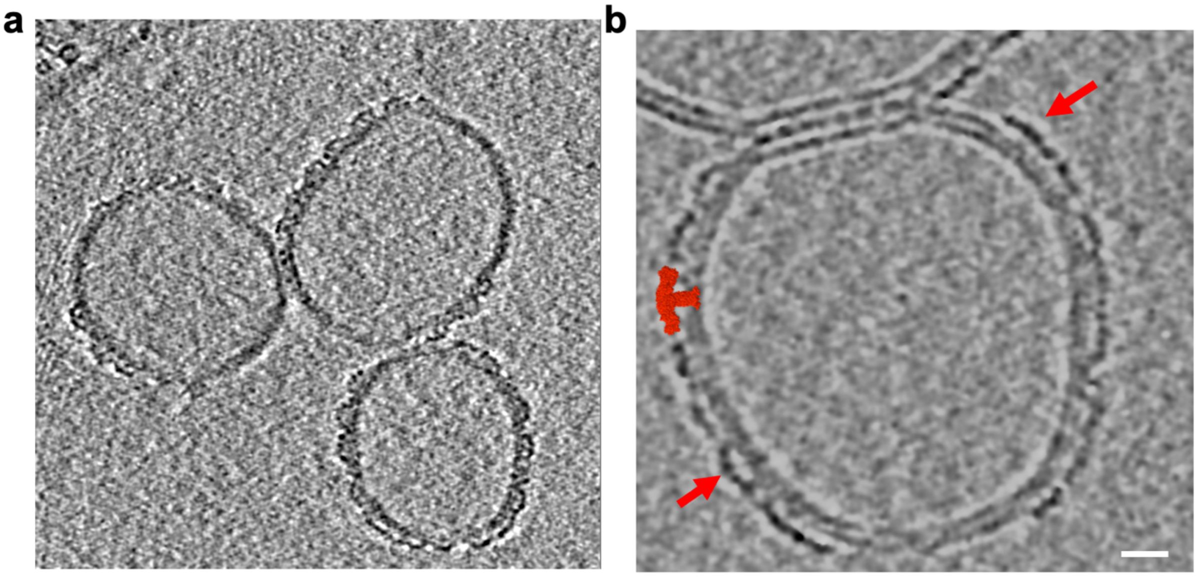

Supplementary Figure 8 : Snapshots of cryogenic electron tomography of aerolysin WT in DOPC:DOPE (2:1) liposomes. a. Overview of aerolysin in liposomes. **b.** Zoom into a liposome. 2 aerolysin WT pores are highlighted by a red arrow and one pore is highlighted in red. The scale bar corresponds to 100 Å (see also Supplementary Video 2).

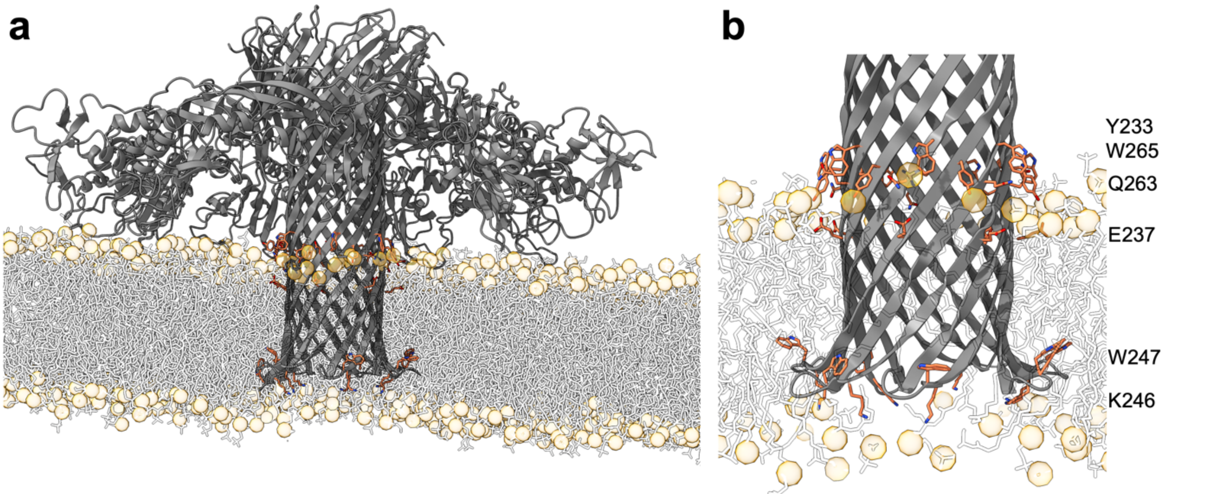

Supplementary Figure 9 : Snapshot of the aerolysin in a DOPC:DOPE (2:1) membrane equilibrated in an atomistic MD simulation, omitting E237 protonation. Phosphate headgroups are highlighted as spheres and the residues Y211, W265, Q263, E237, W247 and K246 are highlighted in orange. The residue E237 is not protonated. **a.** View of the entire protein. **b.** Zoom into the transmembrane barrel region

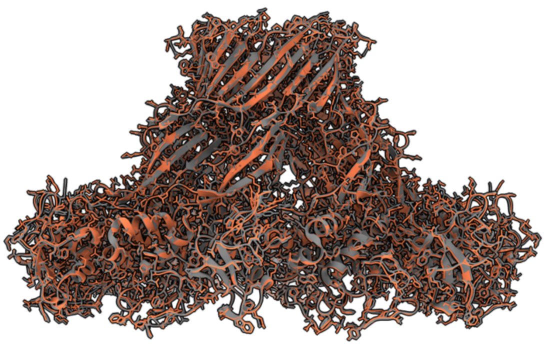

Supplementary Figure 10 : Aerolysin mutant Y221G trapped in its prepore state. In orange the new high-resolution structure is shown. In gray the previous PDB structure (5JZH).

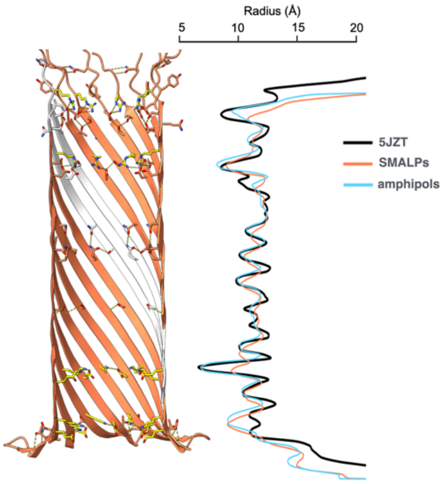

Supplementary Figure 11 : Water accessibility radius for aerolysin WT. Sideview of WT aerolysin lumen in SMALPs. Amino acids that form interactions on the side chain level in the pore lumen are shown as sticks. Amino acids causing constriction are highlighted in yellow .The water accessibility radius across the pore is calculated from MD simulations for the aerolysin WT structure 5JZT, WT in SMALPs and WT in amphipols.

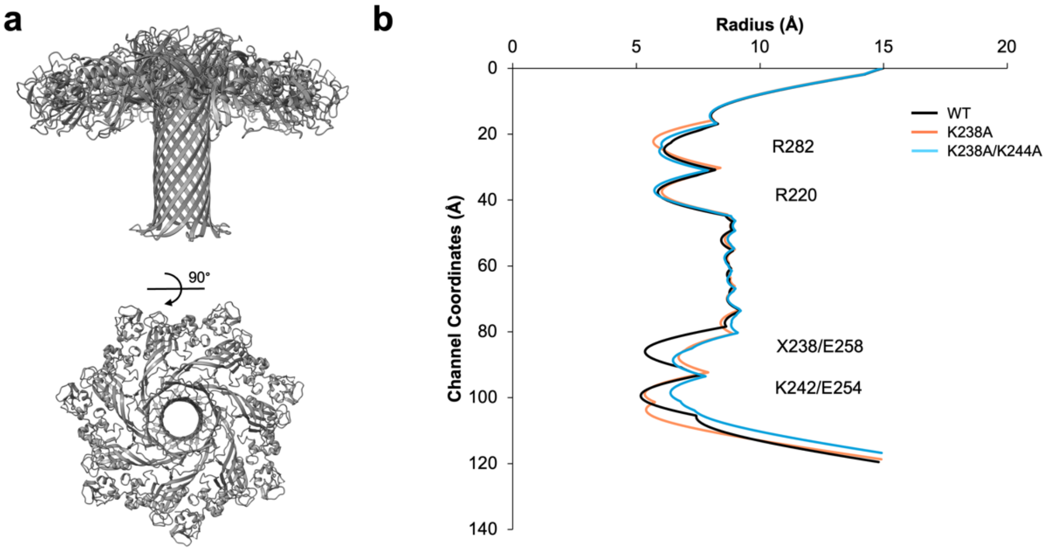

Supplementary Figure 12: Aerolysin K238A/K244A mutant. **a.** Cryo-EM structure of the aerolysin mutant K238A/K244A in SMALP from the top and the side. **b** Comparison of the pore radii for the aerolysin wt (black), K238A mutant (orange) and the K238A/K244A mutant (blue).

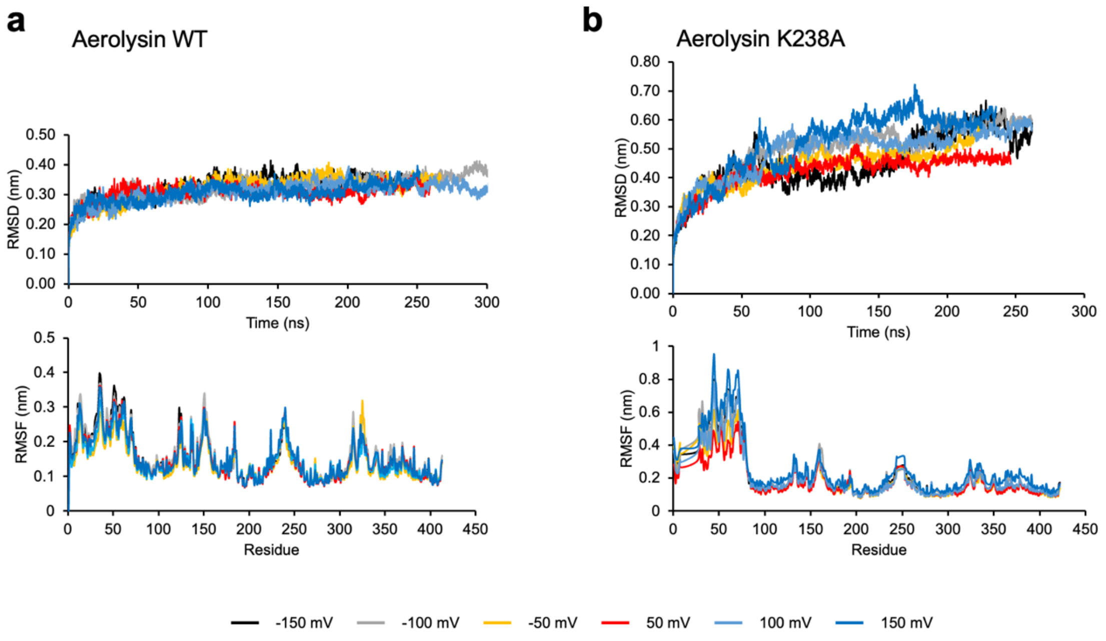

Supplementary Figure 13 : RMSD over the duration of the simulations and RMSF per residue of the all-atom MD simulation of aerolysin WT and K238A and different voltages. a. RMSD over the duration of the simulation (top) and RMSF per residue (bottom) for aerolysin WT. **b**. RMSD over the duration of the simulation (top) and RMSF per residue (bottom) for aerolysin K238A. Simulations at -150 mV (black), -100 mV (gray), -50 mV (orange), -0 mV (red), 100 mV (light blue) and 150 mV (dark blue).
